# Web-based information on the treatment of the mouth in systemic sclerosis

**DOI:** 10.1186/s41927-020-00160-5

**Published:** 2020-11-24

**Authors:** Ismail Abdouh, Stephen Porter, Stefano Fedele, Nadia Elgendy, Richeal Ni Riordain

**Affiliations:** 1 Oral Medicine, UCL Eastman Dental Institute, University Street, London, WC1E 6DE UK; 2 Oral Medicine, College of Dentistry, Taibah University, Al Munawarah, Al Madinah Saudi Arabia; 3 Oral Theme UCLH/UCL NIHR, Biomedical Research Centre, London, UK; 4 Faculty of Dentistry, King Abdulaziz University, Jeddah, Saudi Arabia; 5 Cork University Dental School and Hospital, University College Cork, Cork, Ireland

**Keywords:** Systemic sclerosis, Online information, Patient education

## Abstract

**Background:**

To categorise the content and assess the quality and readability of the web-based information regarding treatment of the mouth in systemic sclerosis.

**Methods:**

An online search using three different search terms regarding the treatment of the mouth in SSc was undertaken using the Google search engine. The first 100 websites from each search were selected for analysis. Data recorded included DISCERN instrument scores along with the Journal of the American Medical Association (JAMA) benchmarks and the presence of the Health on the Net seal (HON). Flesch Reading Ease Scores, Flesch-Kincaid Grade Level, the Simplified Measure of Gobbledygook Index and Coleman-Liau index were calculated to assess readability.

**Results:**

Fifty seven of the first websites remained for analysis after applying appropriate exclusion criteria. The mean overall DISCERN score was 2.37 (±1.01). Only 4 websites (7%) achieved all four JAMA benchmarks. Only 12 websites (21.1%) displayed the HON seal. The reading level was found to be difficult to very difficult among the majority of websites.

**Conclusion:**

The overall quality of the available online information concerning the treatment of the mouth of systemic sclerosis is questionable and requires a high level of reading skill. Further efforts should be directed toward establishing higher quality, reliable online information sources on the treatment of oral disease relevant to patients with systemic sclerosis.

## Background

Systemic sclerosis (SSc) is a rare multi-systemic autoimmune disease that can give rise to a spectrum of manifestations that affect the skin and internal organs [1]. The prevalence of SSc is estimated to be 1–15/100000 of the population affecting middle to late age with more predominance among females [2]. Recent studies have reported that SSc patients have high mortality ratio with survival rates 16–34 years less than the sex- and age-matched controls, due to their active disease and internal organ involvement [3]. Affected individuals often have a disease that may negatively impact upon a patient’s quality of life [4]. About 80–90% of patients with SSc manifest a variety of orofacial features that include fibrosis of the facial skin, microstomia, salivary gland dysfunction (and resultant xerostomia), dysphagia as well as a potential increased risk of caries, periodontal disease and oral malignancy. The extra-oral and intra-oral manifestations of SSc can be challenging to manage effectively and can limit oral function, negatively impact upon facial aesthetics and adversely affects a patients’ emotional and social life [5].

The recent emphasis on shared decision making in a clinical setting places an increased importance upon patient education [6]. To effectively participate in clinical decisions regarding their healthcare patients need to be familiar with the risks and benefits of treatment options being considered [7]. The Worldwide Web is considered one of the most rapidly growing sources of healthcare information and patient self-education. Although such online information is easily accessible and plentiful, there are concerns regarding the poor quality, inaccuracy and difficult readability of health-related information [8]. Thus, online information could be misleading or inaccurate and hence hinder informed shared clinical decision making [9, 10]. In addition, poor quality information can limit the ability of a patient with chronic illness to cope with their disease [11].

Due to the chronic and sometimes progressive nature of the disease, individuals with SSc are likely to require or wish to have the appropriate knowledge to help them to cope with the impairments of the disease. Individuals with oral and/or facial disease of SSc are likely to search for information concerning the features of the disease, their treatment options and perhaps the complications of therapy [4, 12]. There is, however, no data on how helpful online information regarding the orofacial aspects of SSc may be for patients (or carers), hence the aim of the present study was to categorise the content and evaluate the quality and readability of the available web-based information concerning the treatment of the oral aspects of SSc.

## Methods

### Search

An online search using the most popular international search engine (Google.com) was conducted in November 2019 using three different search terms (“Treatment of the mouth in scleroderma”; “Treatment of the mouth in systemic sclerosis”; “Treatment of the mouth in scleroderma/systemic sclerosis”). Although fewer than 25% of people search beyond the first page of a Google search, we included the initial 100 websites in the study to ensure a thorough evaluation of the available online information [13]. The first 100 websites of each term were assessed for duplications and screened for any non-operative link. The following exclusion criteria were then applied; scientific articles, book reviews, websites with non-related content, non-working links, non-English language links, membership-based websites, promotional product websites, discussion groups, video feeds and online medical dictionaries.

The remaining websites were categorised as defined by Ni Riordain and McCreary (2009), based upon affiliation (commercial, non-profit organisation, university/medical centre and government), specialisation (exclusively or partly related to treatment of the mouth in scleroderma/SSc), content type (medical facts, clinical trials, question and answers and human interest stories) and content presentation (image, video and audio).

### Quality assessment

The quality of the online material was assessed independently by two reviewers (IA and RNR) using the DISCERN instrument [14], and the Journal of the American Medical Association (JAMA) benchmarks for website analysis [15]. Training was provided for each reviewer prior to data extraction and any disagreement was resolved by a third reviewer (SRP). The presence of the HON seal was also recorded.

The DISCERN instrument developed and validated to examine the reliability of online content and its specific information on treatment options and overall quality scoring. This instrument was originally developed in the University of Oxford and it consists of 16 items. Questions 1–8 explore reliability, questions 9–15 refer to specific details of information on treatment with an additional question to allow an overall rating of the quality of the material being evaluated. Each question is rated on a numerical scale from 1 to 5 (1 = very poor, 2 = poor, 3 = moderate, 4 = good, 5 = excellent) [14].

The JAMA benchmarks were used to analyse the quality of websites. These benchmarks include clarity of authorship of medical content including (authors, contributors, affiliations and relevant credentials), inclusion of attributions (references and sources), statements of disclosure (ownership, conflicts and interest) and indication of currency (dates of content posted and updates) [15]. Health on the Net (HON) is a non-profit organisation established in 1995 to guide in the evaluation of the reliability of online information and sources in the medical field. The HON seal can be displayed on websites that comply with eight elements ranging from the indication of authors’ qualifications to clearly distinguishing advertising from editorial content.

### Readability assessment

Readability is defined as the determination by systematic formulae of the reading comprehension level a person must possess to understand written texts [16]. The readability assessment was undertaken by using 4 different measures: Flesch Reading Ease Scores (FRES), Flesch-Kincaid Grade Level (FKGL), the Simplified Measure of Gobbledygook (SMOG) Index and the Coleman-Liau index (CLI). The FRES is based upon a formula that incorporates the average sentence length and the average number of syllables per word and the outcome score is a number ranging from 0 to 100. The higher the score - the easier the passage is to read [17]. For example, scores above 90 are considered easily understandable by an average 5th-grade student while scores between 60 and 70 are supposed to be easily readable for 8th and 9th-grade students. Finally, scores less than 50 represented an academic grade level and considered as difficult level of readability.

The FKGL improves upon the FRES and is based on the average number of words per syllables and sentence. The SMOG index takes into account the number of polysyllabic words per sentence and estimates the years of education a person needs to understand a piece of writing. The CLI relies on characters instead of syllables per word and sentence length.

All FKGL, SMOG index and CLI output a U.S. school grade level; this indicate the average student in that grade level can read the text. For example, a score of 7.4 indicates that the text is understood by an average student in 7th grade. However, it has been recommended that FKGL and SMOG index should be 5 or below to be easily comprehended.

Readability was assessed independently by two reviewers (IA and NE). To indicate the textual comprehension difficulty of a text, the following automated formula was used through a website (www.readabilityformulas.com).

### Statistical analyses

Standard descriptive statistical analysis was performed by using SPSS (version 25) and tabulated as mean ± standard deviation of the mean.

## Results

### Available websites

**Fig. 1:**
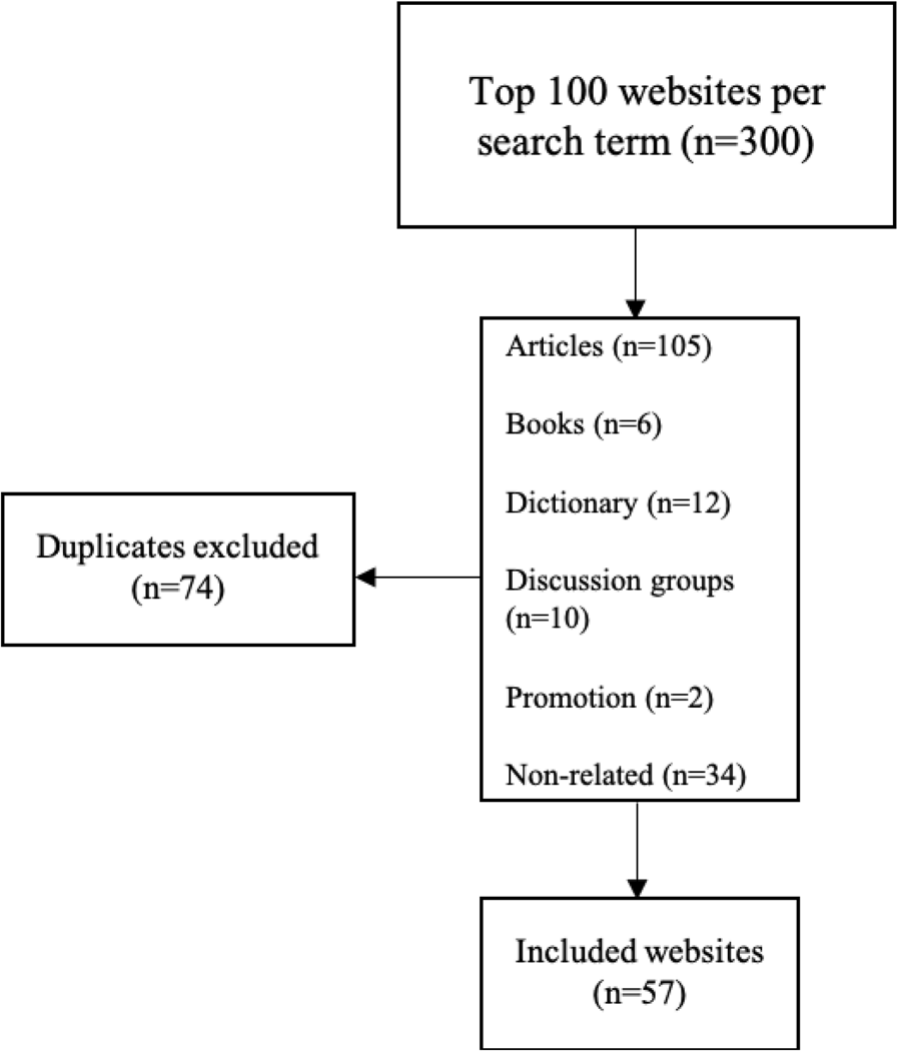
Flowchart of the sample selection strategy

The search strategy for the term “treatment of the mouth in scleroderma/systemic sclerosis” generated 432,000 websites, 440,000 websites for “treatment of the mouth in scleroderma” and 338,000 by searching “treatment of the mouth in systemic sclerosis” on the Google search engine. Of the first 300 websites of the three search terms, 105 were scientific articles, 6 were book reviews, 12 were online medical dictionaries, 10 were links of online discussion groups, two were commercial and 34 were non-related websites. Only 57 selected websites remained for final review after eliminating the duplicates between the three search terms (Fig. 1). With regard to specialisation, it was not possible to determine the exact proportion of each site dedicated to the treatment of the mouth due to the site design and multiple linkages available. However, among these selected 57 sites only 16 sites (28.1%) exclusively dedicated to the treatment of mouth in SSc and 41 (71.9%) were partly related to the treatment of the mouth in SSc.

Regarding the affiliation of the websites, 25 websites (43.9%) were commercial, 23 (40.4%) were non-profit websites, 5 (8.8%) were considered as governmental, and only 4 (7%) were either universities or hospitals. The majority of the websites (82.5%) included medical facts. However, 10 (17.5%) of the sites included clinical trials, 6 (10.5%) included human-interest stories and only 5 (8.8%) included questions and answers. The content presentation varied as 17 (29.8%) websites included images and only one website (1.8%) included an audio illustration. None of the websites included videos. (Table 1) provides a summary of website categorisation.

**Table 1:** Categorisation of websites based on affiliation, specialisation, content type and content presentation

Category	Criteria	Number of websites (%)
Affiliation	Commercial	25 (43.9)
	Non-profit organisation	23 (40.4)
	Governmental	5 (8.8)
	University/medical centre	4 (7.0)
Specialisation	Exclusively related	16 (28.1)
	Partly related	41 (71.9)
Content type	Medical facts	47 (82.5)
	Clinical trials	10 (17.5)
	Human interest stories	6 (10.5)
	Question and answer	5 (8.8)
Content presentation	Image	17 (29.8)
	Video	0 (0)
	Audio	1 (1.8)

### Quality assessment

The mean overall DISCERN score across the 57 selected websites was 2.37 (± 1.01). No website achieved the maximum rating and 13 (22.8%) received the minimum overall rating. The majority of the websites had scores that ranged between 2 and 3. The questions with the poorest DISCERN scores related to the effect of no treatment (“Does it describe what would happen if no treatment were used?”), additional sources of support or information (“Does it provide details of additional sources of support and information?”) and the explicit date of the material published (“Is it clear when the information reported in the publication was produced?”) with mean scores of 2.16 (± 0.75), 2.25 (± 1.5) and 2.26 (± 1.28) respectively (Table 2). Only twelve of the 57 websites (21.1%) displayed the HON seal.

**Table 2:** Means and standard deviation scores for DISCERN

Domain	DISCERN question	Mean (SD)
Reliability	Q1. Explicit aims	2.30 (±0.865)
	Q2. Aims achieved	2.74 (±0.768)
	Q3. Relevance	3.79 (±0.840)
	Q4. Explicit sources	2.46 (±1.377)
	Q5. Explicit date	2.26 (±1.289)
	Q6. Balanced and unbiased	2.61 (±0.701)
	Q7. Additional sources	2.25 (±1.550)
	Q8. Areas of uncertainty	2.74 (±0.992)
Treatment options	Q9. How treatment works	2.61 (±1.013)
	Q10. Benefits of treatment	2.72 (±1.048)
	Q11. Risk of treatment	2.39 (±0.940)
	Q12. Effects of no treatment	2.16 (±0.751)
	Q13. Effects on quality of life	2.81 (±0.953)
	Q14. All alternatives described	3.47 (±1.002)
	Q15. Shared decision	2.74 (±0.720)
Overall rating		2.37 (±1.011)

With regard to the JAMA benchmarks, the majority of the websites (71.9%) fulfilled the authorship benchmark and nearly half of the websites (54.4%) achieved the attribution benchmark. However, only 24 (42.1%) websites achieved the currency benchmark and only 15 (26.3%) achieved the disclosure benchmark (Table 3).

**Table 3:** Websites content based on JAMA benchmarks

JAMA benchmarks	Number	(%)
Authorship	41	71.9
Attribution	31	54.4
Disclosure	15	26.3
Currency	24	42.1

### Readability

FRES ratings varied from 7.48 to 54.18 with mean total readability score of 37.5 (±8.7). FKGL was ranged from 6 to 51 with mean total readability score of 12.5 (±6.4), SMOG was ranged from 6 to 34 with mean total readability score of 11 (±4.2) and CLI was ranged from 5 to 19 with mean total readability score of 13.2 (±2.7).

**Fig. 2:**
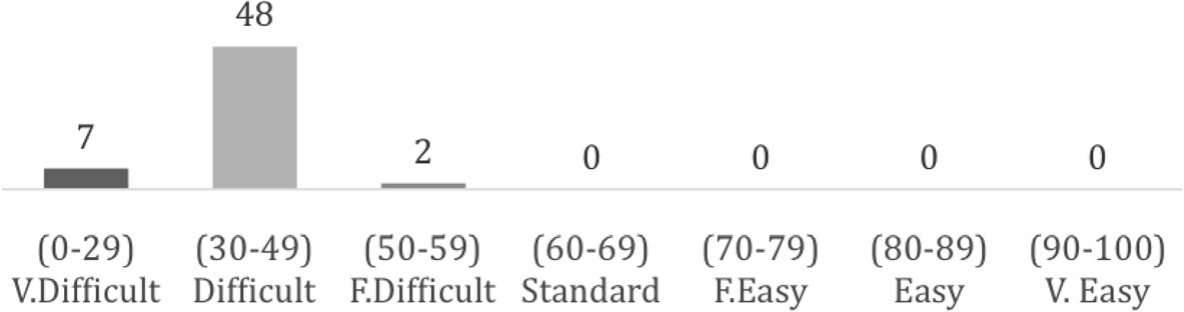
The number of websites per reading easiness grade (*n* = 57)

Based on the four readability measures the majority of the websites (*n* = 55) had readability levels ranging from difficult to very difficult while only two websites had readability level as fairly difficult (Fig. 2).

## Discussion

Patients with chronic diseases such as those managed in a rheumatology setting use the world wide web to seek health-related information more than other groups of patients [9, 12, 18, 19]. As SSc is a chronic disease, which may lead to physical impairment and morbidity, patients commonly search for online information in relation to the disease itself and available therapies [20]. Up to 70% patients with SSc commonly experienced a broad range of symptoms of the disease such as fatigue, Raynaud’s phenomenon, joint pain and muscle pain that might negatively impact upon the health-related quality of life [2]. In addition, SSc can give rise to a variety of orofacial features such as skin fibrosis, microstomia, increase the susceptibility to dental caries and periodontal disease, xerostomia and pathological bone resorption all of which can have a negative impact on patients’ oral health-related quality of life [1]. According to van der Vaart et al., about 85% of patients with SSc use the internet to seek information regarding their condition, with 58–63% of these patients search for information specifically about treatment options and lifestyle management. Patients with SSc use the Internet more frequently and spend more time searching for disease-related information than other patients’ groups, such as those with autoimmune rheumatic diseases [12, 19]. In a recent systematic review of online material for individuals with Raynaud’s phenomenon and SSc, the authors discussed the established need for high quality online information in patient with SSc to aid in the management of the clinical and psychosocial aspects of their condition. They also highlighted the merits of assessing the quality of online information using not only DISCERN but also tools such as HON and JAMA benchmarks [21]. Due to the aforementioned extensive oral and peri-oral manifestation of SSc and the almost ubiquitous use of the Internet as a source of medical information by this patient cohort it is crucial to evaluate the quality and readability of information available online regarding the treatment of the mouth in patients with SSc.

When considering the content of the websites reviewed in this study only 28.1% of the examined websites were exclusively dedicated to the treatment of the mouth in SSc while 71.9% were considered partly related to the treatment of the mouth in SSc. Of note, 82.5% of the sites identified in this study contained medical facts. With over four-fifths of the material being deemed as medical facts, it is unsurprising that the readability level in this study ranged from “difficult” to “very difficult”. Patients searching for material specifically dedicated to the management of the oral manifestations of SSc will not only have to delve into the website content to find content pertaining to the oral cavity but will also have to try to interpret the extensive medical content. Concern has previously been expressed regarding the ability of patients to accurately interpret medical information [22]. Ayonrinde highlights that although the access to high-quality specialist medical texts online is beneficial to the medical community in the pursuit of the practice of evidence-based medicine, the general public lacks the crucial appraisal skills to appreciate the quality of the published material or interpret the data provided. In other studies that have evaluated online health information seeking behaviour of patients with chronic or debilitating diseases a number of barriers have been reported, which include patients being unable to find specific information and an inability of patients to evaluate the material found [23] [24] [25]. Based on the findings of this study, these barriers may be relevant to patients with oral manifestations of SSc, therefore, providing guidance to this cohort of patients on easily accessible and comprehensible online information pertaining to the management of the oral manifestations of SSc is worthwhile.

An alternative means of providing material that is easily understood to patients is to use human-interest studies or patient-based vignettes. These vignettes contain medical content but use lay terminology and present the material often using the patient voice. These human-interest vignettes have been reported to be considered as a form of social and emotional support fo patients [26]. Hay et al. reported that up to 9% of patients attending a rheumatology clinic searching online trying to find people with matching disease features and experiences [27]. In spite of the merits of this form of patient information, only 10.5% of the websites reviewed in this study contained human-interest studies. With the permission of patients under their care, a collaborative initiative between Rheumatologists and Dental Practitioners could provide a series of vignettes could be included in online material, thereby eliminating the need for critical appraisal skills needed for medical texts and providing a form of emotional support for patients with the oral manifestations of SSc.

In considering the reliability of the online material the overall mean DISCERN score of the assessed websites was 2.37 (± 1.01), indicating that the quality of the available information was low to moderate [14]. Similar results have been reported among several studies dealing with different oral health-related conditions [28–30]. These poor results in the overall DISCERN score were mirrored in the study findings for the JAMA benchmarks. Only 7% of the websites met the full JAMA benchmarks while the highest number of websites (30%) achieved two benchmarks. Less than half of the websites achieved the currency benchmark (42.1%), and only 26% achieved the disclosure benchmark while almost half of the sites achieved the attribution benchmark (54.4%).

Recent similar findings were seen across different online sources dealing with other oral health-related conditions and the absence of such information could be considered to be suspicious since patients cannot trust these online sources [11, 30, 31]. The American Food and Drug Administration (FDA, 2005) suggested that the highest quality of online information is usually administrated by governmental, non-profit and academic institutions however the current results showed variations of quality among these available online sources and it might be related to a potential commercial bias as the highest number of included websites were categorised as a commercial sites (43.9%).

## Conclusion

This study highlights the poor quality and questionable reliability of the content of the associated online sources in relation to the treatment of the mouth in SSc. However, when considering the significant impact of SSc upon both physical and psychological aspects of patients, it is worrying that more high-quality patient centred material is not available to those searching online. Current results also suggest that the readability level of the available online information did not meet the recommended levels to be read and understood easily by the general population. Thus at present patients with SSc who are seeking health-related online information should be aware of the substantial unmet needs regarding the available information about the treatment of the mouth and its related conditions. Based on the results of this study, further work is required to ensure high quality, comprehensible and relevant online content is accessible to patients with SSc. In agreement with Devgire et al., we would recommend that this further work would incorporate an assessment of the scientific or clinical accuracy of the material online to promote information that is in concordance with established clinical practice guidelines [21, 32].

## Data Availability

Data available on request from the corresponding author.

## Abbreviations

SSc: Systemic sclerosis
JAMA: Journal of the American Medical Association
HON: Health on the Net
FRES: Flesch reading ease score
FKGL: Flesch-Kincaid grade level
SMOG: Simplified measure of Gobbledygook
CLI: Coleman-Liau index
